# Acute mercury poisoning: a case report

**DOI:** 10.1186/1471-227X-10-7

**Published:** 2010-03-19

**Authors:** Sezgin Sarikaya, Ozgur Karcioglu, Didem Ay, Aslı Cetin, Can Aktas, Mustafa Serinken

**Affiliations:** 1 Dept. of Emergency Medicine, Yeditepe Univ, School of Medicine, Istanbul, Turkey; 2 Dept. of Emergency Medicine, Acibadem Univ, School of Medicine, Istanbul, Turkey; 3 Dept. of Emergency Medicine, Pamukkale University, School of Medicine, Denizli, Turkey

## Abstract

**Background:**

Mercury poisoning can occur as a result of occupational hazard or suicide attempt. This article presents a 36-year-old case admitted to emergency department (ED) due to exposure to metallic mercury.

**Case Presentatıon:**

A 36-year-old woman presented to the ED with a three-day history of abdominal pain, diarrhea and fever. One week ago her daughter had brought mercury in the liquid form from the school. She had put it on the heating stove. One day later, her 14-month old sister baby got fever and died before admission to the hospital. Her blood pressure was 134/87 mmHg; temperature, 40.2°C; heart rate 105 bpm and regular; respiration, 18 bpm; O_2 _saturation, 96%. Nothing was remarkable on examination and routine laboratory tests. As serine or urinary mercury levels could not be tested in the city, symptomatic chelation treatment with N-acetyl cysteine (NAC) was instituted with regard to presumptive diagnosis and history. At the 7^th ^day of admission she was discharged without any sequelae or complaint. At the discharge day blood was drawn and sent for mercury levels which turned out to be 30 μg/dL (normal range: 0 - 10 μg/dL).

**Conclusion:**

Public education on poisoning and the potential hazards of mercury are of vital importance for community health.

## Background

Acute and chronic mercury exposure represents a potential threat to community health. Mercury poisoning can occur as a result of occupational hazard or suicide attempt. Mercury is silver-colored and liquid at room temperature. Mercury is available in inorganic and organic forms. All compounds of mercury are toxic but differ in the routes of absorption, clinical findings, and responses to therapy. Methylmercury, the soluble form is neurotoxic. Elemental (organic) mercury is especially hazardous for children since it is in liquid form and can easily be found around [1].

The clinical effects of mercury poisoning depend on the form and the route of entry to the organism. Neurologic, gastrointestinal and renal systems are predominantly affected depending on the route of exposure.

This article presents a 36-year-old case admitted to emergency department (ED) with nausea, vomiting and diarrhea caused by accidental inhalation and skin exposure of metallic mercury.

## Case Presentation

A 36-year-old woman presented to the ED with a three-day history of abdominal pain, diarrhea and fever. One week ago her daughter had brought mercury in the liquid form from the school without permission from her teacher. She had played with the mercury, and then put it on the heating stove and watched its vaporization. Meanwhile, her mother breast-fed her 14-month old sister. 24 hours after this event her baby got fever and died before admission to the hospital, without any specific diagnosis. The autopsy report disclosed a suspected mercury poisoning which might have led to cardiorespiratory collapse resulting in death of the infant.

On examination, her blood pressure was 134/87 mmHg; temperature, 40.2°C; heart rate 105 bpm and regular; respiration, 18 bpm; O_2 _saturation, 96% with pulse oximetry at room temperature. The patient had no past medical history. Her fever relieved after administration of 1 gr paracetamol given via intravenous route, while arterial oxygen saturation rose to 98% with supplemental oxygen.

Nothing was remarkable in her head-neck, respiratory, cardiovascular, or abdominal examinations. Neurological examination did not reveal any tremor, paresthesia, ataxia, spasticity, hearing and vision loss. Neuropsychiatric abnormalities were not identified.

Complete blood count, urinalysis, sodium, potassium, blood urea nitrogen (BUN), creatinine, aspartate aminotransferase (AST), alanine aminotransferase (ALT), bilirubin levels were within normal ranges. Chest X-ray and cranial computed tomography revealed no findings of disease.

As serine or urinary mercury levels could not be tested in the city, symptomatic chelation treatment with N-acetyl cysteine (NAC) was instituted with regard to presumptive diagnosis and history. At the 7^th ^day of admission she was discharged without any sequelae or complaint. In the same day, blood was drawn and sent for mercury levels which turned out to be 30 μg/dL (normal range: 0 - 10 μg/dL in accord with the hospital laboratory reference). Her symptoms guided the treatment and her laboratory results took three days to be officially reported.

A week after the discharge the patient revisited the ED due to recurrent abdominal pain. Physical examination and laboratory test results were unremarkable and she was discharged after 24-hour observation. Follow-up was scheduled for one week later. In follow-up visit the patient was asymptomatic without any clinical finding. Therefore, NAC treatment was terminated after 14 treatment days. The other children did not exhibit any manifestations of the disease.

## Conclusion

Children are always attracted to elemental mercury with its bright gray appearance [2]. The compound has a short half-life in the blood due to rapid distribution into body compartments. Half life in the body is only two months. Almost all of the absorbed amount is excreted via urination [3]. Mercury is used for the manufacturing of industrial chemicals, paints, explosives, batteries, thermometers, sphygomanometers, electronic instruments, etc. Different mercury compounds are used as antiseptic and diuretic in medicine [1]. It is also an ingredient in the drug Thiomersal which is used to prevent contamination of vaccines.

Acute inhalations of mercury vapors can cause pneumonia, adult respiratory distress syndrome, progressive pulmonary fibrosis and death. Also elemental (metallic) mercury can readily pass to systemic circulation via alveoli present in mercury vapor or directly through the skin. It is also known to pass directly from nursing mothers to infants via breast milk [4]. Predominance of gastrointestinal symptoms and historical findings suggest intoxication with elemental mercury in the present case.

All kinds of neurological findings can be seen in chronic mercury exposure. Some effects of high dose mercury inhalation are shown on Table 1[4,5]. A recently published recommendation guideline stresses that "if the elemental mercury was recently heated (e.g., from stove top, oven, furnace) in an enclosed area, all people within the exposure area should be evaluated at a healthcare facility due to the high risk of toxicity (Grade C)" [1].

**Table 1:** Effects of high dose mercury inhalation [5].

**Central nervous system**	Weakness, unconsciousness, headache, irritability, fatigue, confusion, insomnia, behavioral disorders, tremor, polyneuropathy, reduction or loss of hearing and vision
**Cardiovascular system**	Tachycardia, arrhythmia, hypertension, less frequently similar symptoms of pheochromacytoma
**Respiratory system**	Cough, dyspnea, chest pain, pulmonary edema
**Urogenital system**	Tubular dysfunction, dysuria
**Dermatological**	Erythema, rash, itching
**Digestive system**	Stomatitis, metallic taste in mouth, abdominal pain, nausea, vomiting, diarrhea, colit
**Liver**	Elevation of liver function tests, hepatomegaly, central lobular vacuolization
**Musculoskeletal system**	Fasciculation, myoclonus, myalgia, tremor

Findings in history played a critical role in the diagnosis in the present case. Inquiry for additional acid, alkali, arsenic, phosphorus or iron ingestion did not yield any suspicious finding. History of exposure to mercury, gastrointestinal symptoms and suspicious death of breast fed baby led us to the presumptive diagnosis of acute mercury poisoning. It can be postulated that in the present case neurotoxicity was prevented by NAC treatment which was instituted empirically based on clinical symptoms and history although blood and urine mercury levels were not determined at the time of admission.

Because brain maturation was not completed in young children and fetuses even a small exposure can be fatal [6]. Death of the previously healthy baby in 24 hours prompts consideration of necrotizing bronchitis, pneumonia or respiratory distress syndrome [7]. Inhalation of mercury by the baby can be thought to be the main reason of death.

Initial treatment is keeping the patient away from the environment and toxic agents. NAC is used for chelation of mercury, due to lack of other treatment options. Basically it binds mercury by its cystein groups [1]. The chelating drugs with worldwide application are dimercaprol (BAL), dimercaprosuccinic acid (DMSA), 2,3-Dimercapropropane-1-sulphonate (DMPS) British Anti Lewisite (BAL) (2.5 mg/kg) is also commonly used in the treatment [1,8].

This case report emphasizes the importance of public education on poisoning and specifically, potential hazards of mercury for preventive community health. Training is recommended for school children and teachers on poisoning with heavy metal compounds.

## Competing interests

The authors declare that they have no competing interests

No financial arrangements exist which may be interpreted as having the potential to bias the outcome of this case.

## Authors' contributions

SS.. Data Collection, Data Interpretation. Manuscript Preparation, Literature Search. OK... Study Design, Manuscript Preparation, Data Interpretation. DA... Data Collection. Manuscript Preparation. AC... Data Collection, Literature Search. CA... Data Collection, Literature Search. MS... Study Design, Literature Search. All authors read and approved the final manuscript.

## Consent section

Written informed consent was obtained from the patients' relatives for publication of this case report.

Mustafa Serinken, MD

## Pre-publication history

The pre-publication history for this paper can be accessed here:

http://www.biomedcentral.com/1471-227X/10/7/prepub

## References

[B1] Caravati EM Erdman AR Christianson G Nelson LS Woolf AD Booze LL Cobaugh DJ Chyka PA Scharman EJ Manoguerra AS Troutman WG American Association of Poison Control Centers. Elemental mercury exposure: an evidence-based consensus guideline for out-of-hospital management Clin Toxicol (Phila) 2008 46 1 21 18167033 10.1080/15563650701664731

[B2] Nakayama H Shono M Hada S Mercury exanthem J Am Acad Dermatol 1984 13 848 52 10.1016/s0190-9622(84)80165-6 6203944

[B3] Fischbach FT A manual of laboratory & diagnostic testing 1992 4 Philadelphia: J.B. Lippincott Company 214 6

[B4] Ford MD Tintinalli JE, Kelen GD, Stapczynski JS Metals and Metalloids: Mercury Emergency Medicine: A Comprehensive Study Guide 1999 5 Newyork, McGraw Hill 1191 93

[B5] Risher JF Amler SN Mercury Exposure: Evaluation and Intervention. In: The Inappropriate Use of Chelating Agents in the Diagnosis and Treatment of Putative Mercury Poisoning Neurotoxicology 2005 26 691 9 10.1016/j.neuro.2005.05.004 16009427

[B6] Goldman LR Shannon MW American Academy of Pediatrics: Committee on Environmental Health. Technical Report: Mercury in the Environment: Implications for Pediatricians Pediatrics 2001 108 197 205 10.1542/peds.108.1.197 11433078

[B7] Tchounwou PB Ayensu WK Ninashvili N Sutton D Environmental exposure to mercury and its toxicopathologic implications for public health Environ Toxicol 2003 18 149 75 10.1002/tox.10116 12740802

[B8] Blanusa M Varnai VM Piasek M Kostial K Chelators as antidotes of metal toxicity: therapeutic and experimental aspects Curr Med Chem 2005 12 2771 94 10.2174/092986705774462987 16305472

